# Immunogenicity Risk Assessment of Biotherapeutics Using an Ex Vivo B Cell Assay

**DOI:** 10.3390/antib14030062

**Published:** 2025-07-22

**Authors:** Kevin M. Budge, Ross Blankenship, Patricia Brown-Augsburger, Lukasz K. Chlewicki

**Affiliations:** Eli Lilly and Company, Lilly Corporate Center Indianapolis, Indianapolis, IN 46285, USA; budge_kevin@lilly.com (K.M.B.); ross.blankenship@lilly.com (R.B.); brown-augsburger_patricia_l@lilly.com (P.B.-A.)

**Keywords:** immunogenicity, B cells, anti-drug antibodies, ADA, antibodies, biotherapeutic

## Abstract

Background/Objectives: Anti-drug antibody (ADA) formation can impact the safety, pharmacokinetics, and/or efficacy of biotherapeutics, including monoclonal antibodies (mAbs). Current strategies for ADA/immunogenicity risk prediction of mAbs include in silico algorithms, T cell proliferation assays, MHC-associated peptide proteomics assays (MAPPs), and dendritic cell internalization assays. However, B cell-mediated responses are not assessed in these assays. B cells are professional antigen-presenting cells (APCs) and secrete antibodies toward immunogenic mAbs. Therefore, methods to determine B cell responses would be beneficial for immunogenicity risk prediction and may provide a more comprehensive assessment of risk. Methods: We used a PBMC culture method with the addition of IL-4, IL-21, B cell activating factor (BAFF), and an anti-CD40 agonist mAb to support B cell survival and activation. Results: B cells in this assay format become activated, proliferate, and secrete IgG. A panel of 51 antibodies with varying clinical immunogenicity rates were screened in this assay with IgG secretion used as a readout for immunogenicity risk. IgG secretion differed among test articles but did not correlate with the clinical immunogenicity rating. Conclusions: This dataset highlights the challenges of developing a B cell assay for immunogenicity risk prediction and provides a framework for further refinement of a B cell-based assay for immunogenicity risk prediction of mAbs.

## 1. Introduction

Biotherapeutics, specifically monoclonal antibodies (mAbs), have become a common modality to treat cancer as well as autoimmune and neurodegenerative diseases [1,2,3]. Monoclonal antibodies are particularly appealing due to their high target specificity, long half-life, and minimized off-target effects [4]. One drawback associated with the clinical use of mAbs is the development of anti-drug antibodies (ADAs). Possible consequences of ADA development include infusion-related hypersensitivity and anaphylactic reactions [4,5], altered pharmacokinetics (PK), and reduced pharmacologic activity through the production of neutralizing ADAs [6,7,8].

Many factors influence the immunogenicity potential of an mAb, including those associated with the mAb or the patient [9]. Factors associated with the mAb that influence immunogenicity include non-human sequences or non-human glycosylation patterns [9,10], T cell epitopes, B cell epitopes, homology with human germline sequences, aggregation prone regions (APRs), and formulation impurities [9,11,12]. Germline sequences refer to the unmutated, inherited immunoglobulin gene segments that serve as the template for antibody variable regions [13]. These sequences are critical in immunogenicity prediction as deviations from the germline—introduced through somatic hypermutation or engineering—can be recognized as foreign by the immune system, thereby increasing immunogenic potential [13]. APRs are short peptide stretches (typically 5–15 residues) with a high propensity to self-associate into β-sheet-rich structures [14,15]. These regions are often hydrophobic and structurally exposed during stress or partial unfolding, conditions that can occur during bioprocessing or in vivo [14,15]. Aggregation can enhance immunogenicity by promoting uptake by antigen-presenting cells and facilitating the presentation of otherwise cryptic epitopes [16]. Together, germline divergence and APRs are key features in computational and experimental frameworks for predicting immunogenicity risk in therapeutic proteins. Patient-related factors that influence immunogenicity include administration route, dose level and frequency, immunocompetence (i.e., concomitant treatment with immunosuppressants or underlying autoimmune disease), human leukocyte antigen (HLA) haplotype, and the drug target [9,17]. Typically, immunogenicity risk prediction has relied on in silico algorithms, dendritic cell (DC) internalization, T cell proliferation, and MAPP assays [18,19,20,21,22]. While these assays can be effective, a B cell assessment is not considered in this immunogenicity risk prediction workflow. This is problematic, as B cells are not only professional antigen-presenting cells, but also secrete antibodies, including ADAs [23,24,25].

In the context of an ADA response, B cells can bind the mAb through their cognate B cell receptor (BCR), recognizing the epitope or B cell epitopes present on the therapeutic protein, subsequently processing the therapeutic into peptides, and presenting the antigenic peptide(s) to T cells [9]. This results in T cell co-stimulation and either a T-independent or T-dependent B cell response [9,24,25]. Most importantly, B cells secrete ADAs, which can alter the PK, neutralize the efficacy of the mAb, or pose safety risks [6,7,8]. B cells play a crucial role in the immune response, and without a B cell assessment, there is a higher risk of a false-negative response to highly immunogenic mAbs, such as anti-tumor necrosis factor alpha (TNF) mAbs or drugs that directly modulate T cell function [26,27,28,29]. Additionally, the current strategy may sometimes result in false positives from other in vitro assays. Therefore, a comprehensive strategy for assessing immunogenicity risk should include assays that evaluate antigen uptake and processing (DC internalization), T cell activation, and ADA secretion by B cells.

As a first step for broader immunogenicity risk prediction including B cell activation, we established a modified PBMC culture that supports B cell maturation, activation, proliferation, and IgG secretion. This culture system was developed by modifying culture conditions previously employed for a T cell proliferation assay using peripheral blood mononuclear cells (PBMCs) [18]. Another important aspect of the modification of the culture conditions was based on the B cell monoculture method described by Su et al. [23]. The addition of IL-4, IL-21, BAFF, and an anti-CD40 agonist mAb in the culture medium was critical for supporting a productive B cell response. We chose to use PBMCs instead of a B cell monoculture, so that other immune cells would be present to more closely resemble an in vivo setting. However, CD8^+^ T cells have been shown to reduce T cell responses in a T cell proliferation assay [18]. Therefore, CD8^+^ T cells were also depleted from this assay to maintain optimal helper T cell function, which may be crucial for T and B cell interactions. CD40 is a member of the tumor necrosis factor receptor (TNFR) superfamily and is expressed on B cells, dendritic cells, and other antigen-presenting cells [30,31]. Engagement of CD40 by its ligand CD40L (CD154), typically expressed on activated T cells, initiates a signaling cascade involving TRAF proteins and downstream kinases such as MAPKs and NF-κB [32]. This interaction is essential for B cell proliferation, survival, isotype switching, and the development of germinal centers [33,34,35]. In our study, CD40 stimulation was employed to mimic T cell-dependent B cell activation in vitro. This approach is well-established for inducing robust B cell responses, including the upregulation of activation markers (e.g., CD80, CD86), immunoglobulin class switching, and enhanced antigen presentation [33,34]. The use of an anti-CD40 agonist monoclonal antibody provided a controlled and reproducible method to activate B cells, allowing us to study B cell-intrinsic responses relevant to our immunogenicity assessments.

Additionally, we tested the suitability of this culture system as an assay for the immunogenicity risk prediction of mAbs with known clinical immunogenicity rates using Immunoglobulin G (IgG) secretion as a primary readout. This B cell assay is simple, rapid, scalable, and is more readily implemented in comparison to complex 3D culture or artificial lymph node methods [36,37]. Given the need for donor PBMCs, this B cell assay is ideally suited as a medium-throughput screening assay. To date, this is the largest number of mAbs screened in an immunogenicity assay focused on B cells, but there was no evidence of association between donor IgG secretion fold change values and clinical ADA incidence/immunogenicity rate using this assay. Future work will focus on improving the predictive ability of the assay.

## 2. Materials and Methods

### 2.1. Materials

The following antibodies were purchased from BioLegend (San Diego, CA): mouse anti-human CD45-AF700 (catalog: 304024), mouse anti-human CD19-PE-Cy5 (catalog: 302210), mouse anti-human IgD-APC-Cy7 (catalog: 348218), mouse anti-human CD80-PE (catalog: 305208), mouse anti-human CD86-PE (catalog: 305406), mouse anti-human CD134-PE (catalog: 350004), and mouse anti-human CD137-PE-Cy5 (catalog: 309808). Additional antibodies were purchased from Becton Dickinson Biosciences (BD Biosciences, Franklin Lakes, NJ): mouse anti-human CD27-BV605 (catalog: 740398), mouse anti-human CD24-BV650 (catalog: 563720), mouse anti-human IgM-PerCP-Cy5.5 (catalog: 561285), mouse anti-human CD138-BV421 (catalog: 562935), mouse anti-human IgG-PE-CF-594 (catalog: 562538), and mouse anti-human CD4-BV421. The mouse anti-human MHCII-FITC antibody was purchased from LSBio (Newark, CA; catalog: C134163). Additional reagents include RPMI 1640 media (Gibco, catalog: 11875101), AIM V media (Gibco, catalog: 12055083), Cellular Technology Limited (CTL) anti-aggregate wash (CTL, Shaker Heights, OH; catalog: CTL-AA-005), CTS serum replacement (Gibco, Waltham, MA; catalog: 2596101), compensation beads (BD Biosciences, catalog: 552843), brilliant stain buffer (BD Biosciences, catalog: 566385), Fc Block (BD Biosciences, catalog: 564220), Zombie Aqua Viability Dye (Biolegend, San Diego, CA; catalog: 423101) and Zombie Yellow Viability Dye (Biolegend, San Diego, CA; catalog: 423103), and the CellTrace Far Red Proliferation kit (Invitrogen, Waltham, MA; catalog: C34564). Anti-CD40 agonist biosimilar antibodies were produced at Eli Lilly and Company, Indianapolis, IN. IBA568 is a biosimilar of Selicrelumab originally developed by Hoffman-La Roche (Basel, Switzerland). IBA569 is a biosimilar of Dacetuzumab originally developed by Seattle Genetics (Bothell, WA), and IBA570 is a biosimilar of APX005M originally developed by Apexigen (San Carlos, CA; US patent: US 2018/0327496 A1). Biosimilars are identical in sequence to a marketed compound and have similar structure and function, but due to complexity in biologic manufacturing, they are not identical to the marketed drug product [38]. All biosimilar molecules were produced at Eli Lilly and Company, Indianapolis, IN, except for Tildrakizumab, which was purchased commercially. Sequences for biosimilarantibodies were obtained from the World Health Organization’s International Nonproprietary Name Repository. Plasmids were then transiently expressed in Chinese Hamster Ovary cells and purified by using MabSelect SuRe resin and cation exchange, and then the antibodies were dialyzed into PBS (Cytiva, Chicago, IL, USA; catalog: 17543803). Sequence confirmation and binding affinity were assessed to confirm the correct biosimilar antibody was produced.

### 2.2. Human PBMC and B Cell Isolation and Culture

Blood was obtained from in-house healthy volunteer donors with informed consent from the Research Biological Donation (RBD) program at Eli Lilly and Company (Indianapolis, IN, USA). Alternatively, frozen peripheral blood mononuclear cells (PBMCs) were purchased from StemCell Technologies (Vancouver, British Columbia, Canada; catalog: 70025) or AllCells (Alameda, CA, USA; catalog: LP, CR, MNC). All specimens from the Lilly RBD were collected under IRB-approved protocols owned by RBD administrators. As PBMCs were isolated from healthy donors or obtained from external vendors, no selection criteria were applied for donors. All experiments were performed on separate donors. Blood specimens from the RBD were obtained in heparin sodium cell preparation tubes, which contain a liquid density medium for separation (BD Biosciences, Franklin Lakes, NJ, USA; catalog: 362753), and centrifuged for 30 min at 1500× *g*. The supernatant containing PBMCs was removed, 30 mL of PEH buffer (1× PBS, 2% human serum, and 1 mM EDTA) was added, and then the cells were centrifuged at 600× *g* for 10 min. This step was repeated two additional times. The cell pellet was then resuspended in 26 mL of PEH buffer, and 1 mL was counted using a Vi-CELL XR Cell Viability Analyzer (Beckman Coulter, Brea, CA, USA). PBMCs were resuspended at 5 × 10^7^ cells/mL and transferred to a 5 mL polystyrene tube for CD8^+^ T cell depletion.

**Figure A1:**
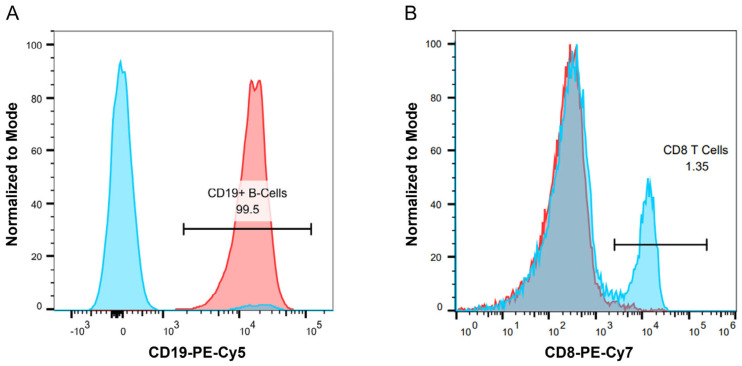
PBMCs were obtained as described in the methods. For B cell monoculture experiments, CD19^+^ B cells were purified prior to culture by using a negative selection kit as described in the methods. For PBMC culture experiments, CD8^+^ T cells were depleted using a positive selection kit as described in the methods. (**A**) Representative histogram plot showing purity for B cell isolation. The blue histogram represents PBMCs without CD19^+^ B cell selection, while the red histogram represents purified CD19^+^ B cells. (**B**) Representative histogram plot for CD8^+^ T cell depletion in PBMC cultures. The blue histogram represents PBMCs without CD8^+^ T cell depletion, while the red histogram represents PBMCs with CD8^+^ T cell depletion.

For both fresh and PBMCs frozen in liquid nitrogen, CD8^+^ T cell depletion was performed using an EasySep human CD8^+^ T cell-positive selection kit II from StemCell Technologies (catalog: 17853). CD8^+^ T cell depletion was assessed by flow cytometry, and cells were used for culture or frozen down in liquid nitrogen for future use. A representative plot of CD8^+^ T cell depletion can be found in Appendix A (Figure A1). For PBMC experiments, cells were counted or incubated in CellTrace Far Red Proliferation Dye (1:1000 dilution in PBS) to measure proliferation for 20 min at 37 °C. If proliferation was not assessed, the CellTrace Far Red Proliferation Dye was not added. Cells were then seeded in 6-, 12-, or 24-well plates depending on the experiment. For six-well plates, the seeding density was 4 × 10^6^ cells per well. For 12- and 24-well plates, the seeding density was 3 × 10^6^ cells per well. No major differences were observed between 6-, 12-, and 24-well plates, so 24-well plates were ultimately chosen for analysis of the 51 mAbs tested in the assay due to the higher throughput. Seeding density and culture plate size are indicated in each figure in the results section. Plates were pre-coated with the IBA570 anti-CD40 agonist mAb, with concentrations indicated in the figure legend for each experiment. Cells were cultured in AIM V medium supplemented with CTS serum replacement (5% final concentration), 10 ng/mL of IL-4 (PeproTech, Rocky Hill, NJ, USA; catalog: 200-04) and 10 ng/mL of BAFF (PeproTech, Rocky Hill, NJ, USA; catalog: 310-13). Additionally, 10 ng/mL of IL-21 (PeproTech, catalog: 200-21) was included in some experiments, as indicated in the figure legend. mAbs tested in the experiments throughout the paper were dosed at a concentration of 0.33 µM (50 µg/mL) except for mAbs 14 and 20, which were dosed at 0.25 µM. mAbs 14 and 20 are in a bispecific format that contained a single-chain variable fragment and thus had a higher molecular weight than a standard mAb. Therefore, 50 µg/mL is equivalent to 0.25 µM for mAbs 14 and 20. A concentration of 0.33 µM has been used previously for a T cell proliferation assay [18]. Cell proliferation was calculated using the following formula: [C/(C + D)]/[A/(A + B)], where A = proliferating untreated cells, B = non-proliferating untreated cells, C = proliferating treated cells, and D = non-proliferating treated cells. This follows what has been previously described for T cell proliferation assays [18,19].

**Figure 1:**
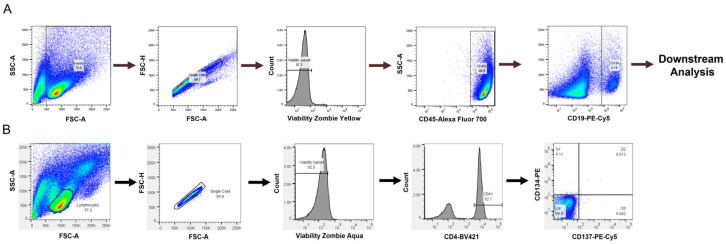
Flow cytometry gating strategy for (**A**) B cell experiments and (**B**) T cell experiments.

**Figure A2:**
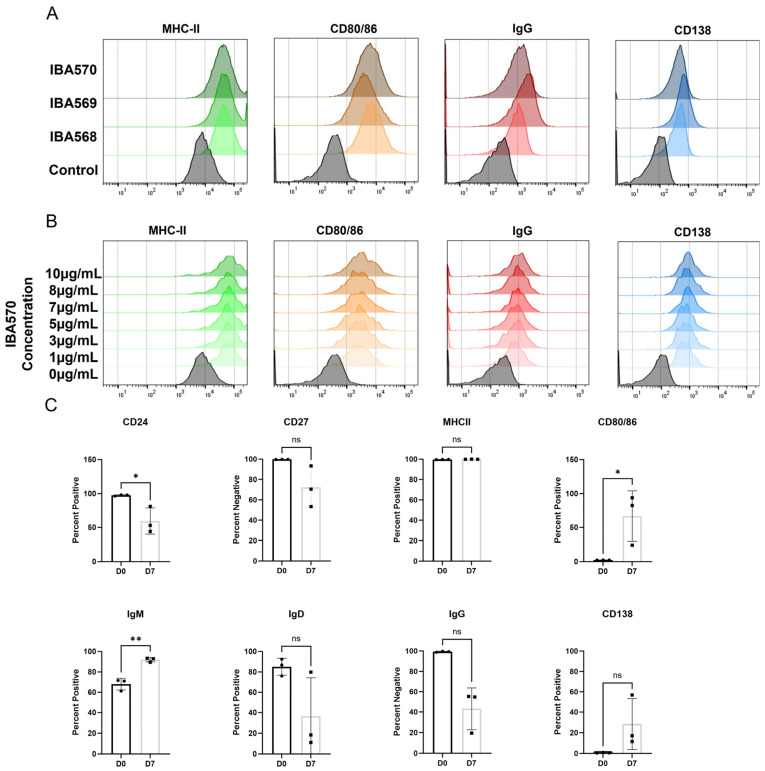
PBMCs were obtained as described in the methods. For B cell monoculture experiments, B cells were purified and cultured for seven days with 10 ng/mL of IL-4, 10 ng/mL of BAFF, and 1–10 µg/mL of anti-CD40 agonist antibody. After seven days B cell surface activation and maturity markers were measured by flow cytometry. (**A**) Representative histogram plots show a comparison between three bead-conjugated anti-CD40 agonist antibodies IBA 568, IBA569, and IBA 570. 5 µg of antibody was conjugated per 1 mg of beads and 0.5 mg of beads were used per 1 × 10^6^ cells. (**B**) Representative histogram plots show a dose response of IBA570 ranging from 1–10 µg/mL. (**C**) B cells were treated with 1 µg/mL IBA 570 for seven days and flow cytometry was performed. * *p* < 0.05, ** *p* < 0.01, ns = not significant.

For the B cell isolation, a human naïve B cell-negative selection EasySep kit from StemCell Technologies was used to obtain naïve B cells (catalog: 19254). Following purification, B cell purity and phenotyping were performed by flow cytometry using single, viable CD45^+^CD19^+^ cells for downstream analysis (Figure 1A). A representative plot of B cell purity from isolations can be found in the Appendix (Figure A2). The naïve B cells were then either used for culture or frozen down for future use. B cells were cultured as previously described [23] with modifications. Briefly, following B cell purification, cells were grown in RPMI 1640 containing 5% human serum, 10 ng/mL of IL-4 (PeproTech, catalog: 200-04), and 10 ng/mL of BAFF (PeproTech, catalog: 310-13) at a density of 250,000 cells/well in 24-well plates. Culture plates were pre-coated with anti-CD40 agonist antibodies with concentrations ranging from 0.1 to 10 µg/mL, as indicated in figure legends. The cell division index (CDI) for B cell proliferation was calculated as described above.

A T cell activation assay was also assessed to determine if a T cell readout could be obtained in our culture conditions, which provide CD40 agonist stimulation to promote B cell survival but not in traditional T cell assays. We used a T cell activation assay developed by Genentech [26]. PBMCs were cultured as described above, and flow cytometry was performed to assess the expression of CD134 and CD137 on CD4^+^ T cells, which are expressed on activated T cells [22]. Lymphocytes were selected from the forward and side scatter plots with additional gating on single, viable CD4^+^ cells to select helper T cells in Figure 1B.

### 2.3. Flow Cytometry

After 7 days of growth in the presence of test antibodies, cells were harvested and centrifuged at 500× *g* for 5 min. The cell pellet was resuspended in 50 µL of Phosphate-Buffered Saline (PBS), and 2.5 µL of human Fc block was added to each sample. Samples were incubated for 15 min on ice, and 50 µL of the B cell staining panel was added to each sample. For each test, the B cell staining panel contained 20 µL of PBS, 10 µL of brilliant staining buffer plus, and 2 µL of each of the following antibodies: CD45, CD19, CD27, CD24, IgM, IgD, IgG, CD138, MHCII, CD80, and CD86. The T cell staining panel contained 20 µL of PBS, 10 µL of brilliant staining buffer plus, and 2 µL each of the following antibodies: CD4, CD134, and CD137. Following incubation with the staining cocktail, cells were washed and incubated for 10–15 min in 100 µL of Zombie Yellow or Zombie Aqua Viability Dye (1:1000 dilution in PBS). Next, 300 µL of flow wash buffer (1× PBS, 0.5% BSA, and 0.1% sodium azide) was added to quench the dye. Samples were then run on an LSR II Fortessa flow cytometer (BD Biosciences). Laser light scatter and fluorescence signals were acquired in biexponential mode. Prior to running samples, single-fluorochrome compensation samples were run to adjust spectral overlap. FCS files were analyzed using Flowjo 10.7.1 flow cytometry software. A biexponential forward scatter (FSC) vs. a biexponential side scatter (SSC) was used to select events. Next, an FSC-A vs. FSC-H bivariate dot plot was used to select singlet cells. A negative gate was used to differentiate between live and dead cells, and only live cells were used for analysis.

### 2.4. Immunoglobulin (Ig) Secretion Analysis

**Figure 2:**
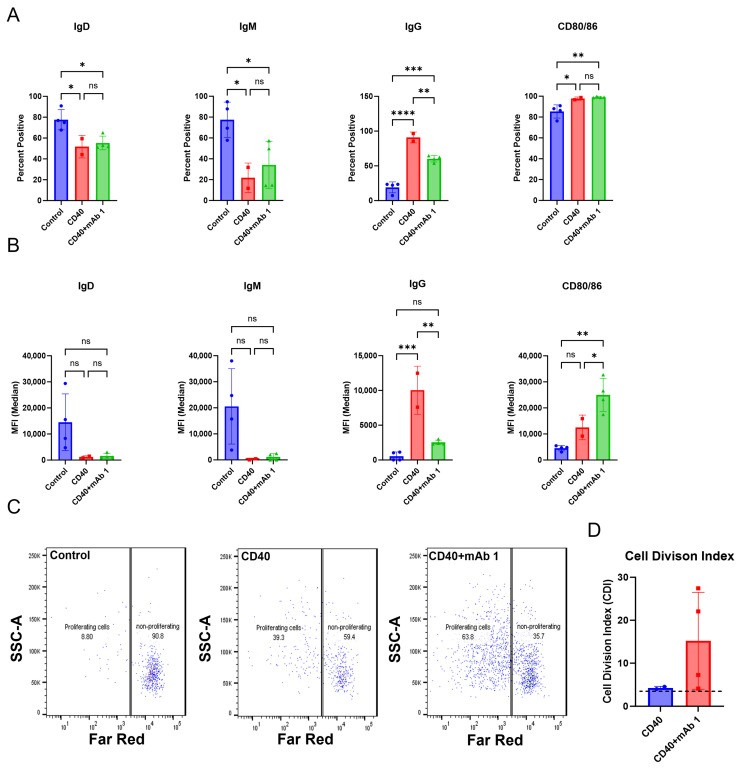

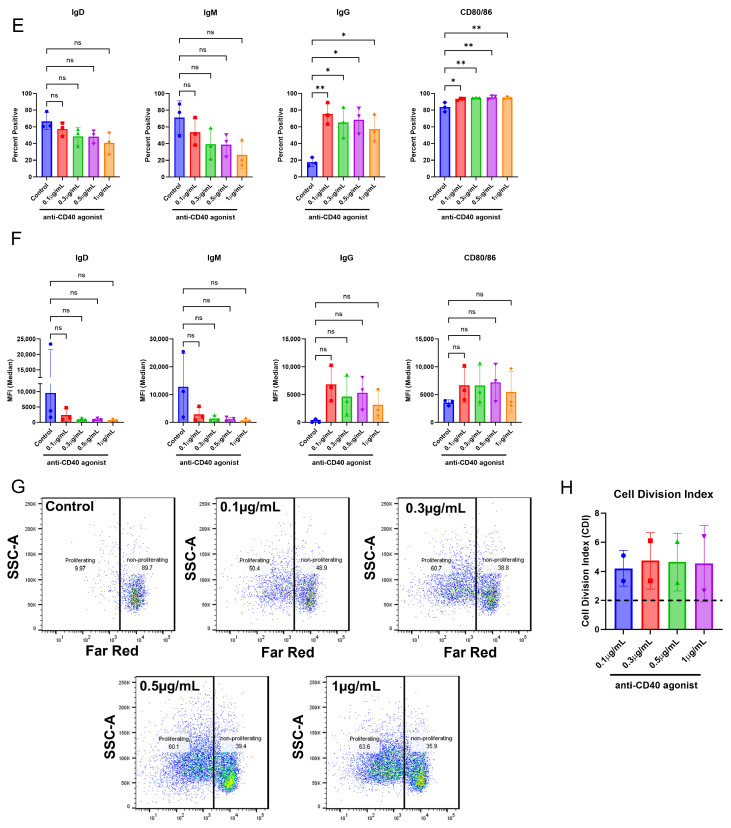

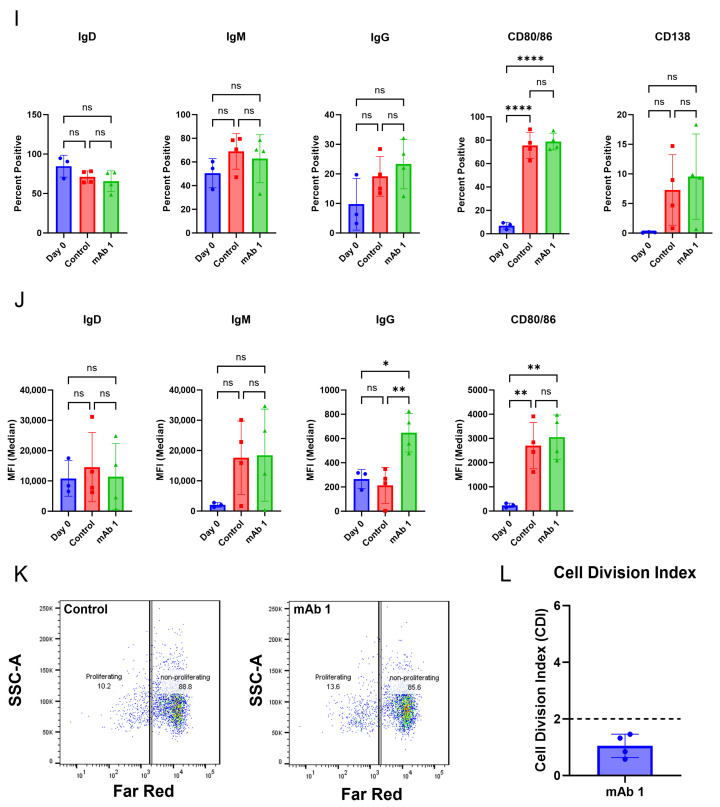
The culture conditions support B cell proliferation and activation. CD8^+^ T cell-depleted PBMCs from two to four donors were seeded into six-well plates at a density of 3 × 10^6^ cells/well with 10 ng/mL of IL-4 and 10 ng/mL of BAFF, and pre-coated with 1 µg/mL of anti-CD40 agonist mAb alone or plus 0.33 µM of mAb 1 and cultured for seven days. (**A**) After seven days, cells were harvested, and flow cytometry was performed to measure the positive cells for membrane IgD, IgM, IgG, and CD80/86 or (**B**) the median fluorescence intensity (MFI) of IgD, IgM, IgG, and CD80/86. (**C**) Prior to culture, cells were incubated with CellTrace Far Red Proliferation Dye, and proliferation was assessed on day seven, with representative plots shown. (**D**) Cell division index after seven days of culture. Next, CD8^+^ T cell-depleted PBMCs from three donors were seeded into six-well plates at a density of 4 × 10^6^ cells/well or 12-well plates at a density of 3 × 10^6^ cells/well and cultured for days with 10 ng/mL of IL-4 and 10 ng/mL of BAFF, and pre-coated with 0.1–1 µg/mL of anti-CD40. (**E**) After seven days, cells were harvested, and flow cytometry was performed to measure the positive cells for membrane IgD, IgM, IgG, and CD80/86 or (**F**) the median fluorescence intensity (MFI) of IgD, IgM, IgG, and CD80/86. (**G**) Prior to culture, cells were incubated with CellTrace Far Red Proliferation Dye, and proliferation was assessed on day seven, with representative plots shown. (**H**) Cell division index following seven days of culture. Next, PBMCs from three donors were seeded into six-well plates at a density of 4 × 10^6^ cells/well and cultured with 10 ng/mL of IL-21, 10 ng/mL of IL-4, and 10 ng/mL of BAFF for seven days with mAb 1. (**I**) After seven days, cells were harvested, and flow cytometry was performed to measure the positive cells for membrane IgD, IgM, IgG, CD80/86, and CD138 or (**J**) the median fluorescence intensity (MFI) of IgD, IgM, IgG, and CD80/86. (**K**) Prior to culture, cells were incubated with CellTrace Far Red Proliferation Dye, and proliferation was assessed on day seven with representative plots shown. (**L**) Cell division index for control or mAb 1-treated cells after seven days of culture. Data are presented as mean ± standard deviation. * *p* < 0.05, ** *p* < 0.01, *** *p* < 0.001, **** *p* < 0.0001. ns = not significant.

Ig secretion was analyzed using the LEGENDPlex Human Immunoglobulin Isotyping Panel (8-Plex) from BioLegend (catalog: 740368). This kit provides quantitation of IgG_1_-IgG_4_, IgM, IgA, IgD, and IgE. Cell culture supernatants were diluted at 1:5 or 1:10 for the assay, and the lower limit of quantitation was 97.6 pg/mL. The upper limit of quantitation was 400,000 pg/mL for the assay. IgG_1_, IgG_2_, and IgG_3_ were calculated by subtracting the fluorescence value from the blank sample, and any negative values after subtraction were set to 0. For IgG calculations in figures following Figure 2, blank subtraction was not carried out due to blank subtraction yielding negative values for some samples. For results greater than the upper limit of quantitation in the assay, a value of 400,000 pg/mL was used for calculations. For results below the lower limit of quantitation in the assay, a value of 97.6 pg/mL was used for calculations. The assay was performed according to the manufacturer’s instructions. Standards and samples were incubated with 25 µL of bead suspension for two hours at room temperature on a shaker and covered with foil to protect them from light. The plates were centrifuged at 250× *g* for five minutes. The supernatant was carefully removed, and the plates were washed with 200 µL of 1× wash buffer. They were then centrifuged again at 250× *g* for five minutes. Samples were incubated with 25 µL per well of detection antibody solution for one hour at room temperature with shaking. After one hour, 25 µL of streptavidin–PE was added to each well and incubated for 30 min. The plates were centrifuged at 250× *g* for five minutes, the supernatant was removed, and the beads were washed with 200 µL of 1× wash buffer. They were centrifuged again at 250× *g* for five minutes. Finally, 150 µL of wash buffer was added to the samples. The plates were analyzed using a high-throughput screener on a BD LSR II Fortessa cytometer (BD Biosciences), and the data were analyzed using FlowJo software. Standard responses were fitted using a 4-PL fit in Prism GraphPad 8.0, and unknowns were determined by interpolation of the standard curve.

### 2.5. Statistical Analysis

All statistical analyses were performed using Prism GraphPad 8.0 except for the ordinal logistic regression results evaluating IgG secretion fold- change over control cultures relative to clinical immunogenicity risk category for the 51 mAbs., This analysis was performed in JMP statistical software (version 17.2.0). A Shapiro–Wilk test was used to test for normality. For comparing two groups an unpaired Student’s T test was performed for data with a normal distribution. A Mann–Whitney test was used for comparing two groups when data did not have a normal distribution. For comparing 3 or more groups, a One-Way ANOVA was performed with a Tukey’s post hoc test for multiple comparisons. A Kruskal–Wallis test with a post hoc Dunn’s test for multiple comparisons was used for data that did not have a normal distribution. A *p*-value of less than 0.05 was considered statistically significant.

## 3. Results

### 3.1. B Cells Become Activated and Proliferate

The B cell monoculture method in Appendix A (Figure A2) was applied to T cell proliferation assay conditions to create a modified system to measure B cell responses. After seven days, cells were harvested for flow cytometry. Anti-CD40 agonist mAb stimulation alone or in combination with mAb 1, which has a high clinical ADA rate of 70%, resulted in decreases in IgD- and IgM-positive cells and median fluorescence intensity (MFI) and increases in IgG- and CD80/86-positive cells and MFI compared to control cultures (Figure 2A,B). Additionally, anti-CD40 agonist mAb stimulation alone or in combination with mAb 1 resulted in robust B cell proliferation compared to control cultures (Figure 2C,D). These changes show that B cells were becoming activated, maturing, and proliferating. However, there did not appear to be differences in activation/proliferation in the anti-CD40 plus mAb 1 compared to anti-CD40 agonist mAb alone cultures, suggesting that the observed activation/proliferation is primarily driven by anti-CD40 agonist mAb stimulation.

Therefore, we determined if we could use lower doses of the anti-CD40 agonist mAb and still achieve an appropriate test-article-related effect on B cells (Figure 2E–H). We found that even with the lowest dose of the anti-CD40 agonist antibody (0.1 µg/mL), IgD- and IgM-positive cells and MFI decreased, while IgG- and CD80/86-positive cells and MFI increased (Figure 2E,F). Furthermore, B cell proliferation was robust even with the lowest doses of the anti-CD40 agonist mAb (Figure 2G,H). The maximal response for most B cell markers and proliferation was observed with the highest dose of anti-CD40 agonist mAb (1 µg/mL), but lower doses provided a similar effect (Figure 2E–H). To assess the potential for mAb 1 to elicit proliferation of B cells in the CD8^+^ T cell-depleted PBMC cultures without the anti-CD40 agonist mAb, cells were cultured at a density of 4 × 10^6^ cells per well in six-well culture plates with 10 ng/mL of IL-21, 10 ng/mL of IL-4, and 10 ng/mL of BAFF. After seven days, cells were harvested for flow cytometry as in previous experiments. It was apparent that activation and proliferation were reduced without anti-CD40 agonism and that the anti-CD40 agonist mAb would be needed for this culture method (Figure 2I–L). Overall, these results suggest that B cell activation and proliferation were minimal compared to previous experiments in which the anti-CD40 agonist mAb was used. Therefore, the anti-CD40 agonist mAb was necessary for B cell proliferation in this culture method. To provide the appropriate stimulus, we proceeded in subsequent experiments with 0.1 µg/mL of anti-CD40 agonist mAb. Control cultures for all experiments moving forward contained 0.1 µg/mL of anti-CD40 agonist mAb and 10 ng/mL of IL-4, IL-21, and BAFF.

### 3.2. B Cells Secrete IgG, Which Is Increased in Response to Treatment with the Immunogenic mAb 1

**Figure 3:**
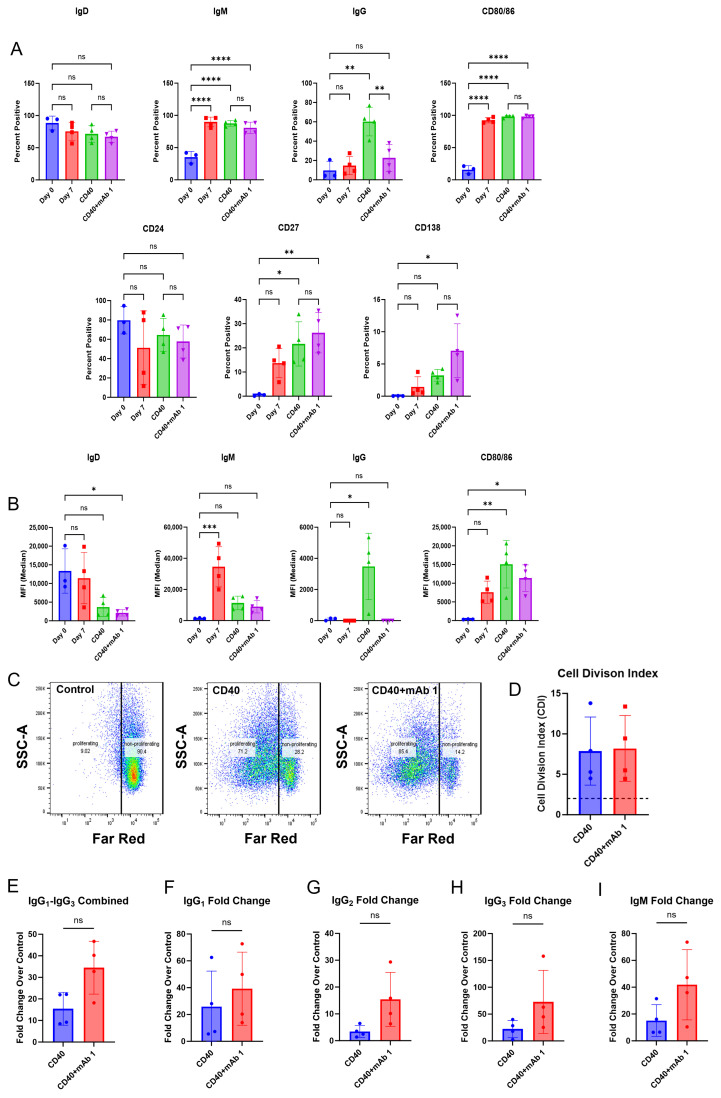
IgG secretion is increased in response to treatment with the immunogenic mAb 1. CD8^+^ T cell-depleted PBMCs from four donors were seeded into 6-well plates at a density of 4 × 10^6^ cells/well or 12-well plates at a density of 3 × 10^6^ cells/well with 10 ng/mL of IL-21, 10 ng/mL of BAFF, and 10 ng/mL of IL-4, and pre-coated with 0.1 µg/mL of anti-CD40 agonist mAb alone or plus mAb 1 and cultured for seven days. (**A**) After seven days, cells were harvested, and flow cytometry was performed to measure the cells positive for membrane IgD, IgM, IgG, CD80/86, CD24, CD27, and CD138 or (**B**) the median fluorescence intensity (MFI) of IgD, IgM, IgG, and CD80/86. (**C**) Prior to culture, cells were incubated with CellTrace Far Red Proliferation Dye and proliferation was assessed on day seven, with representative plots shown. (**D**) Cell division index for control or mAb 1-treated cells following seven days of culture. (**E**–**I**) Additionally, culture supernatants were taken on day seven, and IgG and IgM secretion were measured. Data are presented as mean ± standard deviation. * *p* < 0.05, ** *p* < 0.01, *** *p* < 0.001, **** *p* < 0.0001. ns = not significant.

Next, we determined if the B cells secreted antibodies in our culture in addition to becoming activated and proliferating. Compared to day-zero controls, day-seven controls had changes in markers associated with B cell activation (Figure 3A,B). Similarly to the results in Figure 2, anti-CD40 agonist mAb stimulation alone or in combination with mAb 1 resulted in B cell activation (Figure 3A,B). However, anti-CD40 agonist mAb stimulation in combination with mAb 1 did not cause marker changes compared to treatment with anti-CD40 agonist mAb alone, except for changes in cell surface IgG, which was higher for the anti-CD40 agonist mAb treatment (Figure 3A,B). Additionally, anti-CD40 agonist mAb stimulation in combination with mAb 1 resulted in robust B cell proliferation compared to control cultures, but this was similar to treatment with anti-CD40 agonist mAb alone (Figure 3C,D). These changes show that B cells became activated, mature, and proliferate in our culture system. However, there did not appear to be differential activation/proliferation with the anti-CD40 agonist mAb plus mAb 1 treatment compared to that with the anti-CD40 agonist mAb alone, suggesting that the observed activation/proliferation is primarily driven by anti-CD40 agonist mAb stimulation. Lastly, anti-CD40 agonist mAb treatment alone increased IgG and IgM secretion compared to control cultures, and treatment with anti-CD40 agonist mAb and mAb 1 resulted in increases in IgG and IgM secretion compared to treatment with anti-CD40 agonist mAb alone (Figure 3E–I). These results were encouraging and suggested that this system may be useful in differentiating between low- and high-immunogenicity-rate compounds based on IgG secretion, and not B cell activation/proliferation.

### 3.3. Memory B Cells May Be Responsible for the Observed IgG Secretion

**Figure 4:**
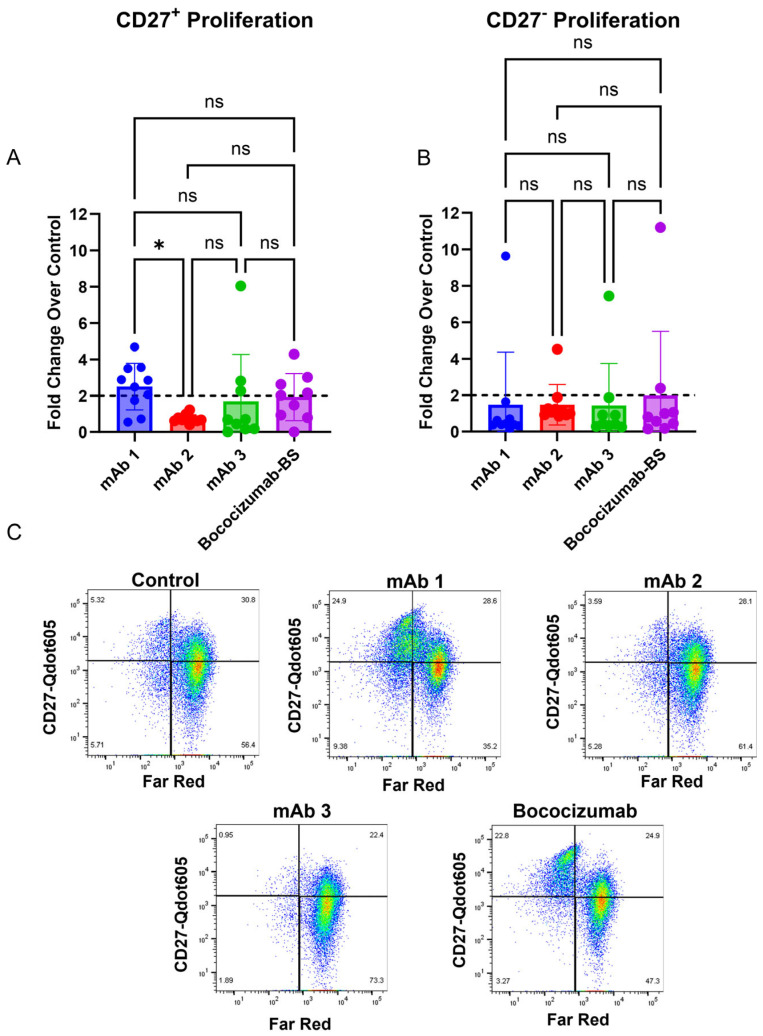
mAbs with high clinical rates of ADAs (mAb 1 and Bococizumab) elicit a stronger CD27^+^ proliferation response compared to mAbs that have low clinical rates of ADAs (mAb 2 and mAb 3 CD8^+^ T cell-depleted PBMCs from 9 to 10 donors were seeded into 24-well plates at a density of 3 × 10^6^ cells/well and cultured for seven days with 10 ng/mL of IL-21, 10 ng/mL of BAFF, and 10 ng/mL of IL-4, and pre-coated with 0.1 µg/mL of anti-CD40 agonist mAb alone or plus test article. After seven days, the culture supernatant was harvested, and flow cytometry was performed. (**A**) The fold change in CD27^+^ B cells following seven days of treatment in the 9–10 donors tested. (**B**) The fold change in CD27^−^ B cells following seven days of treatment in the 9–10 donors tested. (**C**) Representative plots showing proliferation of memory (CD27^+^) and non-memory B cells (CD27^−^). Data are presented as mean ± standard deviation. Dashed lines in (**A**,**B**) represent a two-fold increase. BS = biosimilar. ns = not significant, * *p* < 0.05. ns = not significant.

Published work from Liao et al. suggests that memory B cells are responsible for secreting antigen-specific antibodies toward their tested mAbs [39]. To determine if memory B cells drove the B cell response to mAbs in this study, we quantified the proliferation of CD27^+^ memory B cells and CD27^−^ non-memory B cells (Figure 4A–C). This analysis showed that the mAbs with high clinical rates of ADAs (mAb 1 has a clinical ADA rate of 70% and Bococizumab biosimilar (BS) has a rate of 48% [40]) had 7/10 and 4/9 donors that had a two-fold or greater proliferation of CD27^+^ memory B cells, respectively (Figure 4A). However, mAbs with lower clinical rates of ADAs (mAb 2 has a clinical ADA rate of 1% and mAb 3 has a rate of 2%) had 0/10 and 3/9 of donors that had a two-fold or greater proliferation of CD27^+^ memory B cells, respectively (Figure 4A). All four mAbs showed only one donor that had two-fold or greater proliferation of CD27^−^ non-memory B cells (Figure 4B). Representative flow cytometry plots are shown in (Figure 4C). 

### 3.4. IgG Secretion and T Cell Activation Can Be Obtained from the Same Donors

**Figure 5:**
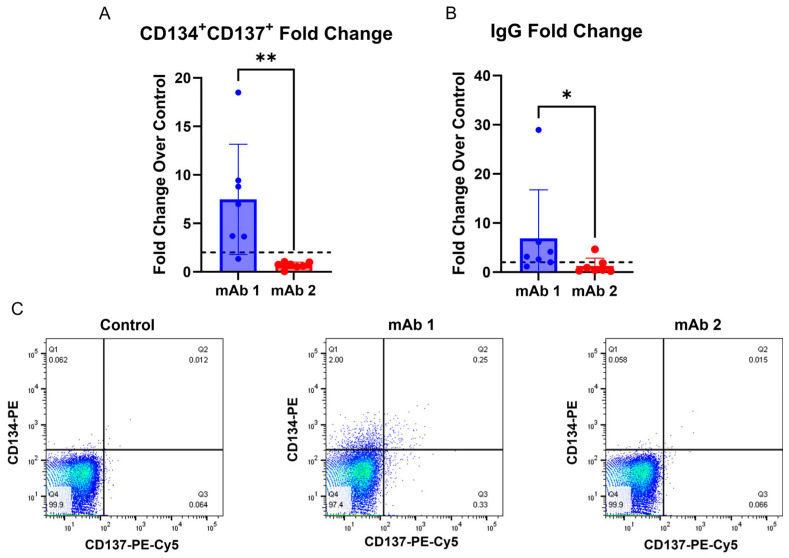
T cell activation and IgG secretion can be obtained from the same donors. CD8^+^ T cell-depleted PBMCs from four donors were seeded into 24-well plates at a density of 3 × 10^6^ cells/well and with 10 ng/mL of IL-21, 10 ng/mL of BAFF, and 10 ng/mL of IL-4, and pre-coated with 0.1 µg/mL of anti-CD40 agonist mAb alone or plus test article for 2 days to measure T cell activation or 7 days to measure IgG secretion. (**A**) After 2 days, cells were harvested, and flow cytometry was performed for CD134 and CD137 expression on CD4^+^ T helper cells. (**B**) After 7 days, culture supernatant was harvested, and IgG secretion was measured using the LEGENDPlex Human Immunoglobulin Isotyping Panel (8-Plex) from BioLegend. (**C**) Representative plots for CD134 and CD137 expression on CD4^+^ T helper cells. Dashed lines in (**A**,**B**) represent a two-fold increase. Data are presented as mean ± standard deviation. ns = not significant, * *p* < 0.05, ** *p* < 0.01.

To determine if T cell activation and B cell IgG secretion can be obtained from the same donor, we utilized a T cell activation assay described previously [26] and performed our B cell assay in parallel. The T-cell activation assay measures the expression of CD134 and CD137 activation markers following two days of culture with an mAb [26]. This assay correlated well with clinical rates of ADAs for the mAbs that they tested. However, our B cell culture method differs from the culture conditions they used in their T cell activation assay. For instance, they did not deplete CD8^+^ T cells, used added cytokines/growth factors, and used human AB serum, whereas we used serum replacement [26]. Thus, it is possible that the T cell activation assay may not work as well in our assay conditions. Following two days of culture, we measured CD134 and CD137 expression on CD4^+^ T helper cells in the cultures treated with mAb 1 or mAb 2 compared to control cultures (Figure 5A,C). We observed an increase in CD134^+^ and CD137^+^ T cells in mAb 1-treated cultures but not mAb 2-treated cultures compared to control cultures. mAb 1 has a high clinical ADA rate (70%), and mAb 2 has a low clinical ADA rate (1%). We also cultured CD8^+^ T cell-depleted PBMCs from the same donors to measure the IgG secretion response. We observed that 6/7 donors treated with mAb 1 had a greater-than-two-fold increase in IgG secretion compared to the control, while only 1/7 donors treated with mAb 2 showed a greater-than-two-fold increase in IgG secretion compared to the control.

### 3.5. IgG Secretion Differs Between Test Articles, but This Does Not Correlate with Clinical Immunogenicity Rates

To test the ability of the CD8^+^ T cell-depleted PBMC assay to differentiate between low-, moderate-, and high-immunogenicity-rate mAbs, we tested a panel of 51 mAbs with known clinical immunogenicity rates in the assay. The panel of mAbs was tested at a concentration of 0.33 µM (50 µg/mL), except for mAbs 14 and 20, which were dosed at 0.25 µM. A concentration of 0.33 µM has been used previously for a T cell proliferation assay [18]. In the IgG secretion analysis, the test mAb remains in the supernatant and provides positive interference for the quantitation of the isotype-specific IgG for the test article. To work around this issue, individual mAb isotypes were quantified in the conditioned media, and stimulation was assessed as the sum of isotypes which were different than the test mAb. For instance, mAb 1 is an IgG_4_ isotype, so the fold change was based on the sum of IgG_1,_ IgG_2_, and IgG_3_. Low-immunogenicity-rate mAbs were defined as when <10% of patients developed ADA, moderate-immunogenicity-rate mAbs were defined as when 10–25% of patients developed ADA, and high-immunogenicity-rate mAbs were defined as when >25% of patients developed ADA. The immunogenicity ratings for the 51 mAbs tested are summarized in Table 1.

**Figure 6:**
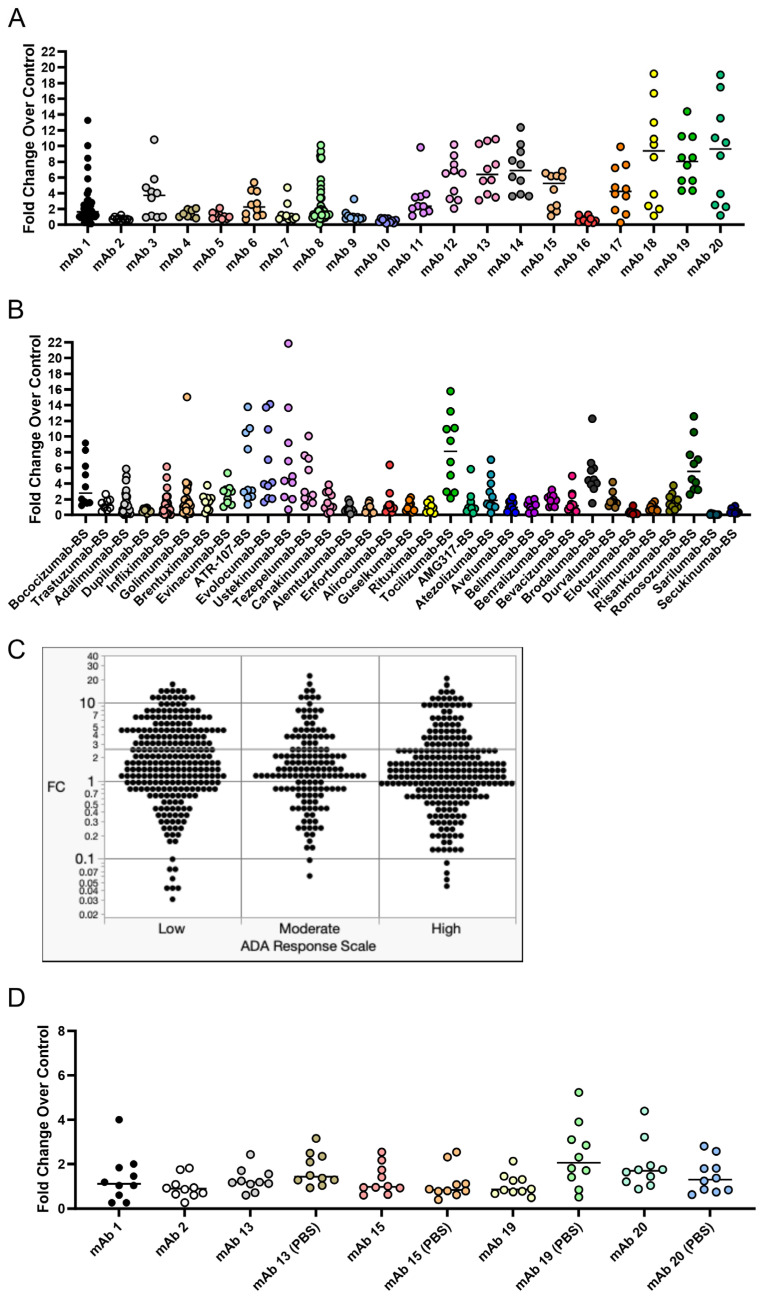
IgG secretion in response to treatment with a panel of 51 mAbs comprising internal and external mAbs with varying clinical immunogenicity rates. CD8^+^ T cell-depleted PBMCs from 8 to 48 donors depending on the mAb (most mAbs were screened using 10 donors) were seeded into 24-well plates at a density of 3 × 10^6^ cells/well with 10 ng/mL of IL-21, 10 ng/mL of BAFF, and 10 ng/mL of IL-4, and pre-coated with 0.1 µg/mL of anti-CD40 agonist alone or plus test article and cultured for seven days. After seven days, culture supernatant was harvested, and IgG secretion was analyzed for all 51 mAbs tested. (**A**) The fold change in IgG secretion compared to control cultures for internal mAbs. (**B**) The fold change in IgG secretion compared to control cultures for external mAbs. (**C**) Visualization of the fold change in IgG secretion compared to control cultures for low-, moderate-, and high-risk mAbs. (**D**) To test if formulation had an impact on the B cell IgG secretion response, we tested mAbs 13, 15, 19, and 20 in their original formulation compared to PBS formulation. Low-risk mAbs were defined as when <10% of patients developed ADA, moderate-risk mAbs were defined as when 10–25% of patients developed ADA, and high-risk mAbs were defined as when >25% of patients developed ADA. BS = biosimilar. Black bars represent the median fold change.

The IgG secretion fold change compared to control cultures for internal mAbs is shown in Figure 6A, while the IgG secretion fold change compared to control cultures for in-house-produced biosimilar external mAbs is shown in Figure 6B. The fold change (FC) of IgG secretion compared to control cultures from each donor treated with low-, moderate-, or high-immunogenicity-rate mAbs is visualized in Figure 6C. Additionally, ordinal logistic regression was performed and is summarized in Table 2 to assess if the fold change in IgG secretion was predictive of a moderate or high clinical immunogenicity rating. The ordinal logistic regression coefficient *p*-value and resubstituted ROC curve areas were examined to assess the potential for any potential association between the distribution of donor fold change values and clinical ADA incidence/immunogenicity rating. There was no evidence of association between donor FC values and the clinical ADA incidence/immunogenicity rate since there were no statistically significant regression coefficients, and ROC curve areas were very near the expected random model level of 0.50. From this experiment, we observed anomalous results that differed from initial experiments. Many low-immunogenicity-rate mAbs elicited a larger magnitude of IgG secretion fold change than we had observed in any of our initial experiments.

This led us to hypothesize that formulation may have an impact on the IgG secretion response in this assay, so we tested a small subset of these mAbs in their original formulation compared to the test article buffer exchanged into PBS (Figure 6D). The original formulation for these subsets of mAbs is listed in Table 3. Interestingly, we observed expected IgG secretion results for mAbs 13 (median fold change of 1.2) and 15 (median fold change of 0.97), which have clinical ADA rates of 13% and 10.2%, respectively. Notably, when mAbs 13 and 15 were originally tested, mAb 13 had a median fold change of 6.4 and mAb 15 had a median fold change of 5.3. We also found that the PBS formulation for mAb 19 caused an increase in the median IgG secretion fold change (0.85 in original formulation vs. 2.1 in PBS), indicating an influence of formulation on the B cell response.

## 4. Discussion

The ultimate goal of in vitro/ex vivo immunogenicity prediction assays is to generate consistent and easy-to-execute readouts from dendritic cells, T cells, and B cells. In this study, we aimed to develop an immunogenicity screening platform that assesses the B cell component during an immune response to an mAb, which is typically not possible with commonly used T cell proliferation assays.

We first replicated the B cell purification and culture as described in [23] and then modified this culture system and applied it to CD8^+^ T cell-depleted PBMC cultures. Within this culture system, B cells were viable, proliferated, and became activated as measured by flow cytometry. However, differences in membrane markers or proliferation did not occur with anti-CD40 agonist mAb plus treatment with the immunogenic mAb 1 compared to anti-CD40 agonist mAb treatment alone. We believe this is due to proliferation and membrane marker changes being driven by anti-CD40 agonist mAb stimulation and the cytokines/growth factors added to the culture, rather than an immunogenic test article. However, we observed a greater-than-two-fold increase in IgG secretion with anti-CD40 agonist mAb plus mAb 1 treatment compared to anti-CD40 agonist mAb treatment alone (Figure 3). This led us to use IgG secretion as the readout for the 51 mAbs we screened in the assay to assess the immunogenicity risk prediction ability of the assay (Figure 6).

Current immunogenicity risk prediction using T cell proliferation assays has some drawbacks. For instance, clinical ADA rates for anti-TNF mAbs, such as Adalimumab, are not well-predicted by T cell proliferation assays [27,28], due to the mechanism of action of anti-TNF mAbs. Furthermore, DC:T cell assays, which are an improvement on standard T cell proliferation assays, still under-interpret the clinical ADA rate of Adalimumab [29]. Interestingly a T cell activation assay developed by Genentech is highly predictive of clinical ADA rates, but similar to other T cell proliferation assays, Adalimumab did not generate a strong response as would be expected based on the clinical ADA rate [26]. These results highlight the opportunity for an assay where Adalimumab and other mAbs that present as false negatives in a T cell proliferation assay format generate a strong response. This would be particularly helpful in the cases where the mAb of interest is an immuno-modulator of T cell activity. The goal of this B cell-based assay was to arrive at a more complete picture of the immune response to immunogenic mAbs and allow for the prediction of traditionally difficult compounds like anti-TNFs. We found that B cell responses, such as activation markers, proliferation, and IgG secretion, can be obtained from our culture method. While we did not see a positive predictive ability of the assay, we believe that further optimization of the assay conditions may yield a positive predictive ability for the immunogenicity risk prediction of mAbs.

Additionally, this method offers the advantage of collecting T cell activation and B cell IgG secretion data from the same donors (Figure 5). Interestingly, donor 3 in the experiment in (Figure 5) was non-responsive in the B cell IgG secretion assay, but responsive in the T cell activation assay following mAb 1 treatment, while donor 4 was responsive in the B cell IgG secretion assay, but non-responsive in the T cell activation assay following mAb 1 treatment. Additionally, donor 2 was responsive in the B cell IgG secretion assay, but non-responsive in the T cell activation assay following mAb 2 treatment. Results in Figure 5 are consistent with clinical ADA rates for mAb 1 and mAb 2 (ADA rates of 70% and 1%, respectively). Providing T cell and B cell readouts from the same donor generates a more complete picture of immunogenicity risk for mAbs and highlights that multiple assays for immunogenicity prediction can complement each other. Further, the use of orthogonal approaches gives the researcher more confidence in human ADA response prediction for compounds of unknown risk.

There is a possibility that the IgG secretion observed in this assay is due to the activation of antigen-specific or cross-reactive memory B cells, rather than the recognition of the test article by naïve B cells and their subsequent class switching to IgG-secreting plasma cells. The B cells were exposed to the culture for seven days, which is relatively early in the window for IgG class switching [41,42]. Other recent work suggests that memory B cells are responsible for secreting antigen-specific antibodies toward a monoclonal antibody [39]. To that end, we observed increased CD27^+^ memory B cell proliferation in response to highly immunogenic mAbs compared to mAbs with low immunogenicity rates (Figure 4). However, we did not observe increased proliferation in CD27^−^ non-memory B cells with highly immunogenic mAbs (Figure 4). Memory B cell proliferation was similar to the IgG secretion results for the four mAbs tested (Figure 6), suggesting that memory B cells, and not naïve B cells, are responsible for IgG secretion in this assay. Future studies will focus on generating a naïve B cell response to mAbs, which may require antigen restimulation or longer culture times given B cell responses in vivo, as previous work has shown that antibody secretion was enhanced in naïve B cell cultures following restimulation after 6 days for an additional 5 days (11 days total) [35]. However, given that we have memory B cells in the culture presented in this manuscript, this process may occur more quickly. To generate a naïve B cell response, future experiments will be performed with CD27^+^ B cell-depleted PBMC cultures. This will also help tease apart whether the IgG secretion is primarily due to memory B cell re-activation or naïve B cell activation for the mAbs we have tested so far.

While developing this assay, we unexpectedly observed a strong response in vitro for several mAbs that have low rates of clinical ADA (Figure 6), which was not observed in our initial experiments. To understand this observation, we explored the impact of formulation on the B cell response. We retested a subset of these mAbs and observed drastically different results for the same mAbs when tested again (Figure 6D). Further work will be needed to improve the repeatability of the assay and determine factors that impact assay response. For instance, a different set of 10 donors was used between Figure 6A and D. While not likely to explain the vast difference in response to mAbs 13 and 15 observed between Figure 6A and D, it is possible that these two different sets of donors behaved differently in the assay. Additionally, this type of assay may need to utilize 30–50 donors to accurately assess immunogenicity, a much larger cohort than is typically used in T cell assays [43]. We also observed that the PBS formulation for mAb 19 caused an increase in IgG secretion compared to its original formulation (10 mM histidine, 15 mM NaCl, 0.02% PS-80, pH 6.0), indicating an influence of formulation on the B cell response can be detected in this assay. Formulation can alter protein conformation [44,45] and impact antigen presentation/recognition. This has been observed for interferon beta in ex vivo T cell assays [44,45]. Overall, we believe that unknown factors (i.e., different donors, formulation, endotoxin levels, aggregation, host cell proteins, or reagent issues) may have contributed to the unexpected results we observed in Figure 6A,D, and further research is needed to better understand the intricacies that may impact assay performance and response.

Interestingly, recent work using both PBMC- and whole-blood-based assays to assess the B cell response to antibodies found that the generation of antigen-specific B cells correlated with clinical ADA rates [46]. As the authors indicate, the whole blood assay had high sensitivity (100%) but low specificity (33%) when using a fold change of 5 as their cutoff for positivity in the assay, highlighting that the best use of the assay is as an added tool to current immunogenicity workflows, and not as a replacement for existing assays [46]. We agree with this point regarding our assay. While we did not find statistical correlation to clinical ADA rates, there may be instances where our assay could complement an existing immunogenicity risk assessment, particularly after further optimization. Additionally, as the authors point out, only 10 molecules for which clinical ADA rates exist were tested, so a wider screen of molecules will be needed to assess the true correlative nature of their assay [46].

In summation, our results indicate that using a modified PBMC assay is sufficient to induce B cell activation and an IgG secretion response from B cells after seven days of culture. The B cell response we observed in this study is likely due to memory B cell activation (Figure 4) and cross-reactive immunity [39]. No single currently established assay can accurately predict the risk of ADA development for all mAbs. Therefore, a combination of in vitro and in silico assessments is performed in the pre-clinical drug development process. With further optimization this B cell assay can be integrated into current immunogenicity risk prediction workflows in pre-clinical drug development. This integration will help in understanding treatment-emergent ADA risk and contribute to the accuracy of these assessments. Further work is also needed to determine the best application of this assay for immunogenicity risk prediction. A recent review suggests that application of this type of assay is best served during the lead selection phase of drug development or in post hoc analysis for ADA-positive patients [43]. Also, optimization of the assay may be needed to enhance its predictive ability and to include the T cell activation assessment.

## 5. Conclusions

Our assay can be used to study the B cell response to mAbs, which is likely driven by memory B cells, but further optimization is needed before the inclusion of this assay into immunogenicity screening workflows. The most promising approach going forward may be to establish a memory B cell-depleted PBMC culture method. This will likely require re-optimization of culture conditions. Despite several low-immunogenicity-rate antibodies generating a strong response in the assay (Figure 6), we believe that this dataset provides important information for the field of immunogenicity research and highlights the challenges of developing a B cell assay for immunogenicity risk prediction of mAbs.

## Figures and Tables

**Table 1:** Clinical immunogenicity risk rating for the panel of 51 mAbs screened in the B cell assay. Low-immunogenicity mAbs were defined as when <10% of patients developed ADA, moderate-immunogenicity mAbs were defined as when 10–25% of patients developed ADA, and high-immunogenicity mAbs were defined as when >25% of patients developed ADA.

Feature	Clinical Risk Rating	Reference for Clinical Rate
mAb 1	High	Internal
mAb 2	Low	Internal
mAb 3	Low	Internal
mAb 4	Moderate	Label (internal)
mAb 5	Moderate	Internal
mAb 6	Low	Label (internal)
mAb 7	Low	Internal
mAb 8	High	Internal
mAb 9	High	Internal
mAb 10	High	Internal
mAb 11	Low	Internal
mAb 12	Low	Internal
mAb 13	Moderate	Internal
mAb 14	Low	Internal
mAb 15	Low	Internal
mAb 16	Moderate	Internal
mAb 17	Low	Internal
mAb 18	High	Internal
Bococizumab-BS	High	[40]
Adalimumab-BS	High	Label
Trastuzumab-BS	Low	Label
Dupilumab-BS	Low	Label
Infliximab-BS	High	Label
Golimumab-BS	Moderate	Label
Brentuximab-BS	Low	Label
Evinacumab-BS	Low	Label
ATR-107-BS	High	Label
Evolocumab-BS	Low	Label
Ustekinumab-BS	Moderate	Label
Tezepelumab-BS	Low	Label
Canakinumab-BS	Low	Label
Alemtuzumab-BS	High	Label
Enfortumab-BS	Low	Label
Alirocumab-BS	Low	Label
Guselkumab-BS	Low	Label
Rituximab-BS	High	Label
Tocilizumab-BS	Low	Label
AMG-317-BS	High	Label
Atezolizumab-BS	High	Label
Avelumab-BS	Moderate	Label
Belimumab-BS	Low	Label
Benralizumab-BS	Moderate	Label
Bevacizumab-BS	Low	Label
Brodalumab-BS	Low	Label
Durvalumab-BS	Low	Label
Elotuzumab-BS	Moderate	Label
Ipilimumab-BS	Moderate	Label
Risankizumab-BS	Moderate	Label
Romosozumab-BS	Moderate	Label
Sarilumab-BS	Low	Label
Secukinumab-BS	Low	Label

**Table 2:** Ordinal logistic regression results which compare the IgG secretion fold change over control cultures to clinical immunogenicity risk category for the 51 mAbs tested in the assay. To quantitatively assess the statistical association between donor fold change (FC) values and the clinical ADA response scale, six different summary statistics of the donor FC values for each test article were computed. For each summary statistic assessed, a univariate ordinal logistic regression was conducted using JMP statistical software (version 17.2.0).

Feature	*p*-Value	Moderate ROC AUC	High ROC AUC
FC median	0.41	0.58	0.58
Proportion donor FC > 2.0	0.35	0.57	0.54
Proportion donor FC > 2.5	0.25	0.60	0.54
Proportion donor FC > 3.0	0.44	0.55	0.49
Proportion donor FC > 3.5	0.35	0.58	0.52
Proportion donor FC > 4.0	0.49	0.57	0.48

**Table 3:** Original formulations for the buffer exchange experiment in Figure 6D.

Test Article	Original Formulation
mAb 13	10 mM histidine, 0.02% polysorbate 80, pH 5.8
mAb 15	10 mM sodium citrate, 25 mM sodium chloride, pH 6.0, 0.005% polysorbate 80
mAb 19	10 mM histidine, 15 mM NaCl, 0.02% PS-80, pH 6.0
mAb 20	10 mM histidine, pH 6.0, 0.006% polysorbate 80

## Data Availability

Data can be made available upon reasonable request from the authors.

## Appendix A

Figure A1 Purity for B Cell Isolation and CD8^+^ T Cell Depletion in PBMC Cultures.

Figure A2 Anti-CD40 Agonist Antibodies Lead to Changes in B Cell Surface Markers Associated with B cell Maturation/Activation.
